# Molecular Characterization and Expression of Lactoferrin Receptor (LfR) in Different Regions of the Brain Responding to Lactoferrin Intervention

**DOI:** 10.1007/s12035-024-04378-z

**Published:** 2024-08-24

**Authors:** Siqi Wang, Nai Zhang, Bowen Jiang, Bo Lönnerdal, Yue Chen, Bing Wang

**Affiliations:** 1 School of Medicine, Xiamen University, Xiamen City, 361102 China; 2 Institute of Process Engineering, Chinese Academy of Sciences, Beijing, 100190 China; 3 Department of Nutrition, University of California, Davis, CA 95616 USA; 4 Gulbali Institute for Agriculture, Water and Environment, Charles Sturt University, Wagga Wagga, NSW 2678 Australia

**Keywords:** Lactoferrin receptor, Lactoferrin supplementation, Working memory, Neurodevelopment, Piglet

## Abstract

Lactoferrin (LF), an iron-binding glycoprotein rich in human milk, promotes neurodevelopment and cognition, but whether it acts through the LF receptor (LfR) and its expression profile in the brain remains unknown. We characterized 972 bp of piglet brain LfR cDNA and found LfR mRNA was expressed all brain regions being highest in the frontal lobe, followed by parietal lobe, brainstem, occipital lobe, cingulate gyrus, subventricular zone, olfactory bulb, hippocampus, amygdala, cerebellum, and thalamus. LfR mRNA and protein in different regions of the brain responded to low (155 mg/kg/day) and high (285 mg/kg/day) LF supplementation of piglets from postnatal days 3 to 38. By postnatal day 39, the low LF diet significantly increased LfR protein expression in the occipital lobe compared to controls, but not the high LF diet. LfR protein in the subventricular zone of the high LF group was 42% and 38% higher than that of the low LF group and controls, respectively. There was a trend for a dose–response relationship between LF intervention and LfR protein expression only in the prefrontal and parietal lobes. LF supplementation significantly improved piglet working memory for a difficult task, which was positively correlated with LfR protein in the prefrontal, parietal, and occipital lobes, but no dose response. Brain LfR responds to dietary LF supplementation, a mechanism by which LF can promote learning and working memory through its receptor. LfR is expressed in the whole brain, and its expression level is anatomic region specific.

**Supplementary Information:**

The online version contains supplementary material available at 10.1007/s12035-024-04378-z.

## Background

Lactoferrin (LF) is a sialylated iron-binding milk glycoprotein with a molecular mass of about 80 kDa and is ~ 10 times higher in concentration in human milk than in cow milk. LF provides several health benefits, including modulating immune responses, regulating iron absorption in the small intestine, and functioning as an antioxidant, anticancer agent and in protection against microbial infection [1–5]. Studies have found that dietary intervention with bovine milk LF (bLF) significantly enhances learning and memory of neonatal piglets by upregulating the brain-derived neurotrophic factor (BDNF) signalling pathway and the expression level of brain polysialic acid, a marker of neuroplasticity, cell migration, and differentiation of progenitor cells, as well as growth and targeting of axons [6]. Maternal bLF supplementation is a beneficial nutritional intervention able to revert some IUGR-induced sequelae, including brain hippocampal changes [7]. Recently, we demonstrated that feeding piglets with a low LF diet (155 mg/kg/day) in pig milk substitute from postnatal days 3 to 38 promotes neurodevelopment and cognitive ability, while feeding a high LF diet (285 mg/kg/day) showed better neuroprotective effects [6, 8]. However, whether brain LfR expression responds to dietary LF and contributes to improved neurodevelopment, neuroprotection and cognition during early development remain unknown.

The multiple biological activities of LF depend on its target cells and on the presence of specific receptors at their surfaces [9]. LF exerts many of its diverse biological functions by binding to its receptors (LfRs). Schryvers et al. reported that the complex LfR in gram-negative bacteria [10], LF-binding protein A (LbpA), and LF-binding protein B (LbpB) appears to be essential for bacterial survival, because these bacteria can specifically bind host LF, e.g., LbpA to the C-lobes of human LF and extract the iron from LF as a source of iron for growth [10, 11]. LfR was first identified in *Xenopus laevis* oocyte and designated XL35 or XCGL-1 [12, 13]. The cDNAs encoding two human homologs of the *Xenopus* oocyte LfR, XL35, were isolated from a small intestine cDNA library and termed hIntL-1 and hIntL-2 [14]. Cox et al. first suggested the presence of a receptor, by which LF donates its iron to intestinal tissue of infants [15]. Due to high homology to human LF, bLF can be taken up by the human small intestinal LfR (SI-LfR) and exert bioactivities [16]. Therefore, commercially available bovine LF is also biologically active. It has been shown that the SI-LfR takes up LF and LF-bound iron into human intestinal epithelial cells [17]. SI-LfR mediates apo- and holo-LF endocytosis via a clathrin-mediated endocytosis pathway in an enterocyte model [18]. Interestingly, in a cytotrophoblast model, LfR mediates apo- but not holo-LF internalization via clathrin-mediated endocytosis [19].

The anatomy and physiology of the piglet digestive system and central nervous system closely resemble those of human infants, making the piglet an excellent animal model for biomedical research on human infants [20, 21]. Recently, we demonstrated that LF upregulates intestinal gene expression of BDNF and UCHL1 as well as alkaline phosphatase activity to alleviate early weaning diarrhea in postnatal piglets [22]. In piglets, SI-LfR expression significantly increases with age in the duodenum and decreases in the jejunum [23]. However, the molecular cloning and functional expression of porcine LfR in different brain regions are largely unknown. Further, no information is available on whether brain LfR expression responds to dietary LF which in turn contributes to neurodevelopment and cognitive function during early life. Therefore, we characterized the distribution and expression profile of LfR in different brain regions and determined how LfR in different regions of the brain respond to different concentrations of dietary bLF.

## Materials and Methods

### Animals

Three-day old male domestic piglets (Durant × Yorkshire × Landrace, ternary cross) weighing 1.6 ~ 2.5 kg were purchased from Xiamen Muxing Industrial Co. Ltd. in Xiamen City, China. Piglets were stratified according to weight and litter and randomly allocated to 1 of 3 treatments. Piglets were housed in pairs in a temperature-controlled room (22–24 °C) with a heat lamp in their home pen on a 12-h light (08:00–20:00) and dark (20:00–08:00) cycle. All animal housing conditions were the same as previously published [8, 24, 25]. The study protocol was approved by the Xiamen University Animal Ethics Committee (Approval Number: AE1640102).

### LF Supplementation and Animal Feeding

Piglets were fed a standard sow milk replacer containing a protein mixture consisting of soy:whey:casein (50%:38%:12%) from 3 to 38 days of age, which is equivalent to a 5–7-month-old human infant based on the brain growth curve as a benchmark [26]. Bovine LF (93% purity and ~ 15% iron saturated, DMV International, The Netherlands) was added to sow milk replacers (Feed & Grow International Co. Ltd. China) at specified concentrations using the following design: 0.06-g LF/L (control group with no added LF, *n* = 16), 0.6-g LF/L (low LF dose, *n* = 17), and 1.1-g LF/L (high LF dose, *n* = 18). These concentrations resulted in an approximate LF intake of 15, 155, and 285 mg/kg body weight/day, respectively. The milk replacers were formulated so that total protein concentration remained the same regardless of the dose of added LF. These doses were chosen because they are similar to the LF levels in sow’s transitional and mature milk and human mature milk, respectively [27]. To maintain normal growth rates, the piglets received 285 ml/kg body weight/day from postnatal day 3 to 15 and 230 ml/kg body weight/day for the remaining 23 days. These levels of dietary bovine LF are less than 15% of the highest safe concentration (2000 mg/kg/day [28]) recommended by the European Food Safety Agency (EFSA) in 2012 (https://efsa.onlinelibrary.wiley.com/doi/pdf/10.2903/j.efsa.2012.2811). The procedure for piglet feeding and health status monitoring during the trial was the same as in our previous publications [6, 22, 25]. Body weight of the piglets was measured each morning before feeding using a digital scale (PRIS-Scale model: XK 3116, Chengdu Pris Electronic Co. Ltd., China). Laboratory staff members who undertook the biochemical assays were blinded to treatment.

### Working Memory Performance Assessment

The working memory performance assessment was carried out at the Pig Behavior Laboratory, Xiamen University, using the validated and published methods [6, 8, 25, 29–31]. To reduce initial stress, both piglets in each pair were allowed into the maze for six habituation sessions to ensure that all piglets could freely open and close the doors of the eight arms 1 day prior to the formal test [6, 8, 25, 29–31].

Formal learning and working memory tests began on day 23 using an eight-arm radial maze adjacent to the home pens. A video camera was installed overhead to record the working memory tests. Two tests were performed: an easy task and a difficult task using our published method as described in the Supplementary Fig. 1 [6, 8, 25]. Both tests had milk accessible (corresponding to their treatment group) in one arm and inaccessible in the remaining seven arms, such that all eight arms of the maze received the same olfactory signals. In both tests, a visual cue consisting of three black dots was placed randomly on a door with milk accessible in the arm. In the easy task, one black dot was placed on the remaining seven doors. In the difficult task, two black dots were placed on the remaining seven doors. The position of the three black dots visual cue was changed between trials in a predetermined random order.

**Fig. 1:**
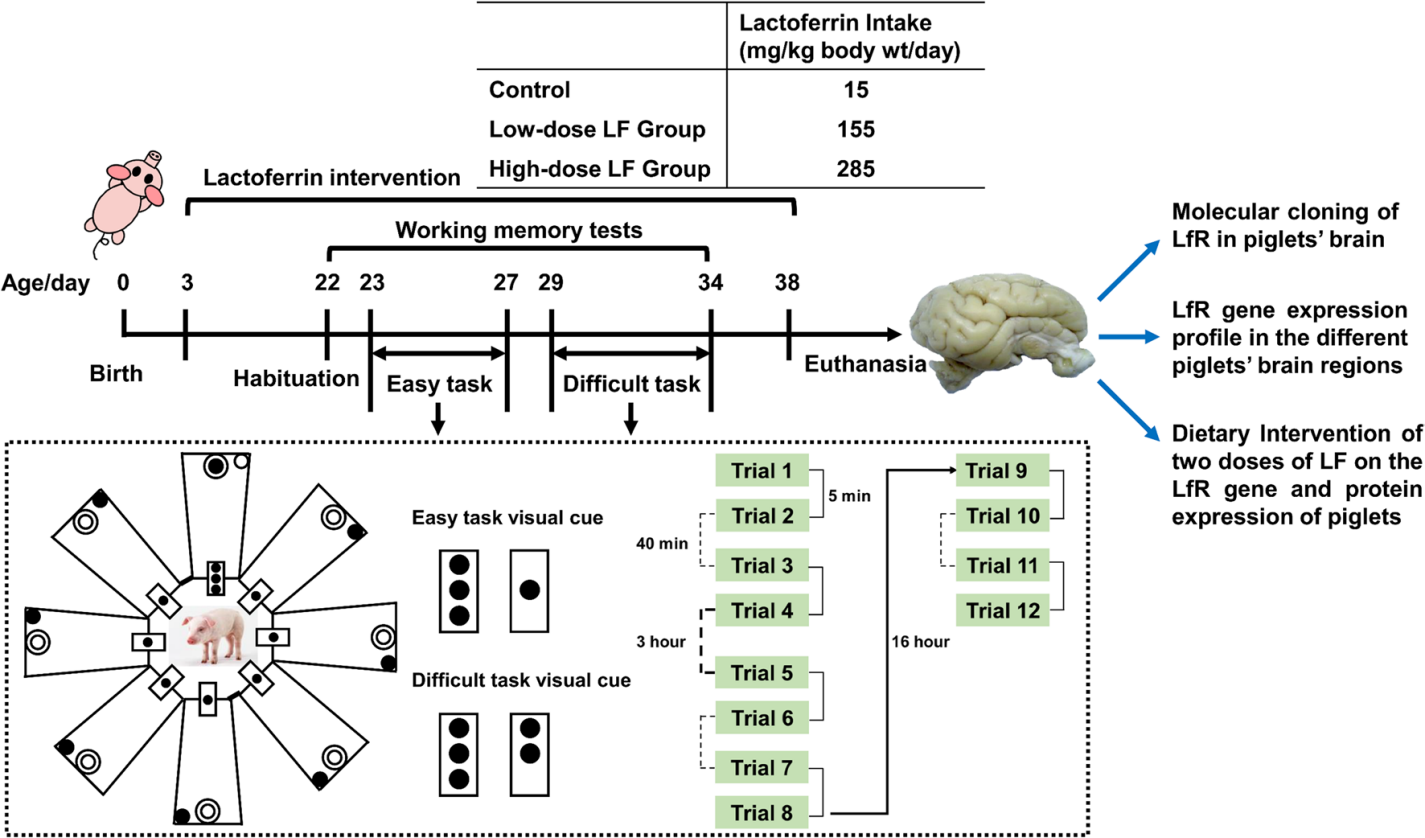
Diagram describing the experimental design

All piglets were tested individually in the maze. Forty trials of the easy task were conducted during 5 days (8 trials/day) and 40 trials of the difficult task during 6 days. Four daily trials were performed in the morning and four trials in the afternoon Each piglet underwent two consecutive trials, with a 5-min interval between to change the visual cues and fresh milk and then returned to the home pen. Forty minutes later, the piglets were returned to the radial maze for another two consecutive trials. In the afternoon, the piglets repeated the morning test protocol. A diagram describing the experimental design and the retention interval after each trial is shown in Fig. 1. A mistake was registered each time the piglet entered or put its whole head through the wrong door. Success was registered only when a piglet entered the correct door and found the accessible milk. Assessment of working memory performance was determined as the number of mistakes (wrong doors) that the piglets repeatedly entered or put its whole head through the same incorrect arm during the trial. All the tests were conducted by a fixed group of trained researchers throughout the trial to reduce the possibility of differences in handling the piglets by different people, which could cause stress or behavioral changes to the piglets. All researchers were blinded to the LF supplementation. Results were corroborated by independent analysis of the video material.

Throughout the trial, all testing was conducted by a fixed group of trained researchers to reduce potential stress or behavioral changes in the piglets caused by differences in the way different people handled the piglets.

### Tissue Collection

**Fig. 2:**
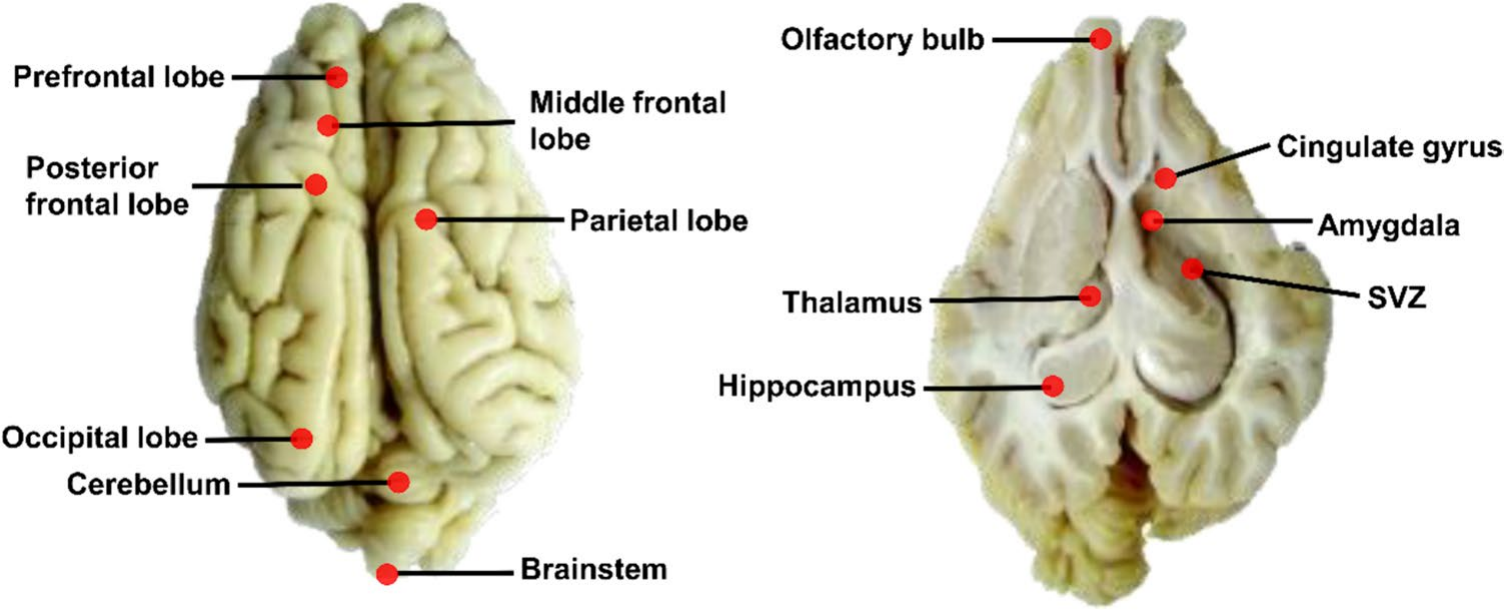
Schematic diagram of the 13 subregions of the piglet brain analyzed in this study. Prefrontal lobe (PF), middle frontal lobe (MFL), posterior frontal lobe (PFL), parietal lobe (PL), brainstem (BS), occipital lobe (OL), subventricular zone (SVZ), cingulate gyrus (CG), olfactory bulb (OB), hippocampus (HIP), amygdala (AMY), cerebellum (CE), and thalamus (THA)

At 39 days of age, the piglets were euthanized. Tissues from different regions of the brain including prefrontal lobe (PF), middle frontal lobe (MFL), posterior frontal lobe (PFL), parietal lobe (PL), brain stem (BS), occipital lobe (OL), subventricular zone (SVZ), cingulate gyrus (CG), olfactory bulb (OB), hippocampus (HIP), amygdala (AMY), cerebellum (CE), and thalamus (THA) were collected after dissection (Fig. 2) [32]. Tissues were sliced stored in RNA safer II® solution (R0424-02, Omega Bio-tek, USA) for PCR experiments and at − 80 °C for subsequent Western blot analyses.

### Cloning of LfR cDNA in the Porcine Brain

LfR cDNA was generated from isolated porcine brain tissue placed in 1.5 ml of RNALater. After removal of RNALater solution, total RNA was extracted from the different regions of brain tissue (100 mg) for molecular characterization of the LfR using a total RNA extraction kit (DP419, Tiangen, China). RNA integrity was evaluated by electrophoresis on a 1.2% agarose gel (ST004K, Beyotime, China). cDNA was generated from 2-μg total RNA using the FastQuant RT Kit with gDNase (KR106, Tiangen, China). PCR primers were designed by Beacon Designer 8 (PREMIER Biosoft, USA) referenced in the reported sequence of LfR (GenBank: AY609994.1) [23, 33]. LfR primer sequences were the sense primer —ATGCCAGCTCAGAGGATTTTT, and antisense primer — TCAGCGGTAGAACAGGAGC.

PCR amplification was performed in 50-μl reaction mixture containing 1-μl cDNA as the template, 0.2-μM antisense primers, 25-μl PrimeSTAR Max Premix (2X) (R040, Takara, Japan), and 22-μl double distilled water. PCR was carried out by initial heating at 98 °C for 1 min, followed by 45 cycles of denaturation at 98 °C for 10 s, annealing at 62 °C for 30 s, extension at 72 °C for 30 s, and a final extension at 72 °C for 10 min. The PCR products were purified by a 0.8% agarose gel (A8201, Solarbio, China) and sent to Sangon Biotech Company for sequencing. DNAMAN 9 software (Lynnon Biosoft, USA) was used to compare the amplified porcine LfR cDNA sequence with the published porcine LfR sequence (AY609994.1) and with the human and mouse LfR sequences.

### Quantitative Real-Time PCR

To quantify expression levels of porcine LfR mRNA in the different brain regions, we used our previously published method [6, 22, 34]. Briefly, mRNA levels of the porcine LfR and reference genes, glyceraldehyde 3-phosphate dehydrogenase (GAPDH), and hypoxanthine phosphoribosyltransferase 1 (HPRT1) were quantified using the ABI 7500 real-time thermocycler (Bio-Rad, USA). Primer sequences and accession numbers for GAPDH, HPRT1, and LfR genes are described in Table 1 cDNA, corresponding to 2000-ng total RNA, served as a template in a 20-μL reaction mixture containing primers and SYBR Green Master Mix (04913850001, Roche, Switzerland). RT-PCR was performed in 20-μl reaction mixtures using the following protocol: 95 °C for 10 min; 40 cycles of 95 °C for 15 s, 60 °C for 20 s, 72 °C for 30 s, followed by a final stage of 95 °C for 15 s and 60 °C for 1 min; and 95 °C for 30 s and 60 °C for 15 s. The standard curve and melt curve for LfR, HPRT1, and GAPDH primers are shown in (Supplementary Fig. 2A, B, C, D, E, F, G, H, I). Relative expression of the LfR gene in different brain regions and LF intervention was calculated as a gene quantity divided by a normalization factor (NF), which was obtained from the geNorm software (version 3.5) (http://medgen.ugent.be/~jvdesomp/genorm/) according to our published method [24, 32]. All analyses were performed in duplicate using our published method [6, 22, 34].

**Table 1:** Primer sequences and accession number used in real-time PCR

Gene	Primer sequences	Products size (bp)	Accession number
LfR	F: 5′-AGCCATTCCTGTTGTCTAT-3′	199	Figure 3A
	R: 5′-GAATCCTCCTCCACCAAT-3′		
GAPDH	F: 5′-GTGAAGGTCGGAGTGAAC-3′	147	NM_001206359
	R: 5′-GGTGGAATCATACTGGAACA-3′		
HPRT1	F: 5′-CCGAGGATTTGGAAAAGGT-3′	181	NM_001032376
	R: 5′-CTATTTCTGTTCAGTGCTTTGATGT-3′		

### Extraction of Total Protein from Porcine Brain

Brain tissue (500 mg) was homogenized in 1 ml of cold HEPES–EDTA buffer (20-mM HEPES, pH 7.4, 1-mM EDTA, 250-mM sucrose/protease inhibitor mixture containing 4-(2-aminoethyl) benzenesulfonyl fluoride, trans-epoxysuccinyl-L-leucylamido (4-guanidino) butane, bestatin, leupeptin, aprotonin, and sodium EDTA, 1 × complete protease inhibitor) (P8340, Sigma, USA) [23]. The homogenate was centrifuged at 5000 g for 10 min at 4 °C. The pellet was collected and resuspended in Tris buffer (2-mM Tris, 50-mM mannitol), incubated for 20 min with 10-mM CaCl_2_ on ice, and then centrifuged at 3600 g for 10 min. The supernatant was collected and centrifuged at 38,000 g for 20 min. The pellet was resuspended in Tris buffer again as described above and centrifuged at 38,000 g for 20 min. The final pellet was resuspended in 250-ml Tris buffer containing 1 × complete protease inhibitor (P8340, Sigma, USA). Protein concentrations were determined using the Bradford assay (Bio-Rad, USA).

### Western Blot Analysis

Twenty micrograms of protein for each sample was subjected to SDS-PAGE electrophoresis and then transferred to a PVDF membrane at 350 mA for 1 h. The membrane was first blocked for 1 h in 1 × TBST containing 10% nonfat milk (10-mM Tris, 150-mM NaCl, 0.05% Tween-20 (S15016, Yuanye, China)) and then incubated with anti-LfR antibody [17, 23] at 1:1000 and anti-β-actin antibody (A1978, Sigma-Aldrich, USA) at 1:1000 at 4 °C overnight. The blots were washed 3 times with 1 × TBST followed by incubation with Goat Anti-Rabbit IgG, (H + L) HRP conjugate (1:50,000, AP124P, Millipore, USA), and Goat Anti-Mouse IgG, (H + L) HRP conjugate (1:50,000, AP132P, Millipore, USA). After a second cycle of washing with 1 × TBST, the immune complexes were detected by enhanced chemiluminescence (CW0049A, CWBIO, China). Finally, the membranes were exposed to X-ray film (XBT-1, Kodak, USA). Quantification of protein band density was performed using Image Analysis Software (National Institutes of Health (NIH), USA).

### Statistical Analysis

Comparisons between means in LfR gene expression profile in each brain region were carried out using general linear model univariate analysis of variance (ANOVA)] with Bonferroni’s adjustment for multiple comparisons. Comparisons of LfR gene and protein expression in different regions of brain, working memory, body weight, and brain weight between the high- and low-dose LF groups and the control group of piglets were performed by a general linear model ANOVA with Bonferroni’s post hoc test for multiple comparisons. The correlations between LfR protein in different regions of the brain and total number of mistakes in working memory of the difficult task were also analyzed using bivariate Pearson correlation coefficients. A correlation was taken to be significant if *p* < 0.01 (2-tailed). The *F*-test was used for the comparison of the slopes of the regression line of LfR protein level in the parietal lobe and occipital lobe against total number of mistakes in the difficult task. Data are presented as mean ± SEM. Values were considered significant at *p* < 0.05. All statistical analyses were completed with the use of IBM SPSS statistics 25 (SPSS, Inc., Chicago, IL, USA).

## Results

### Characterization of Porcine Brain LfR cDNA

**Fig. 3:**
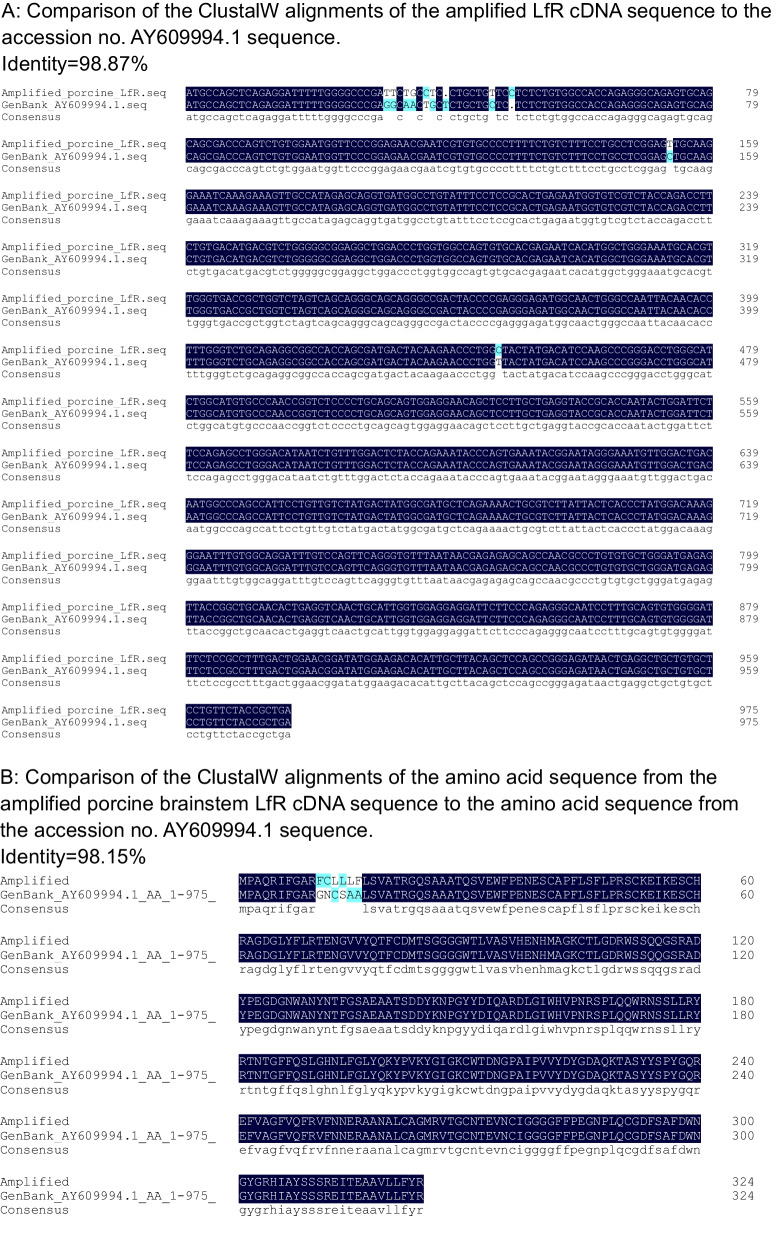

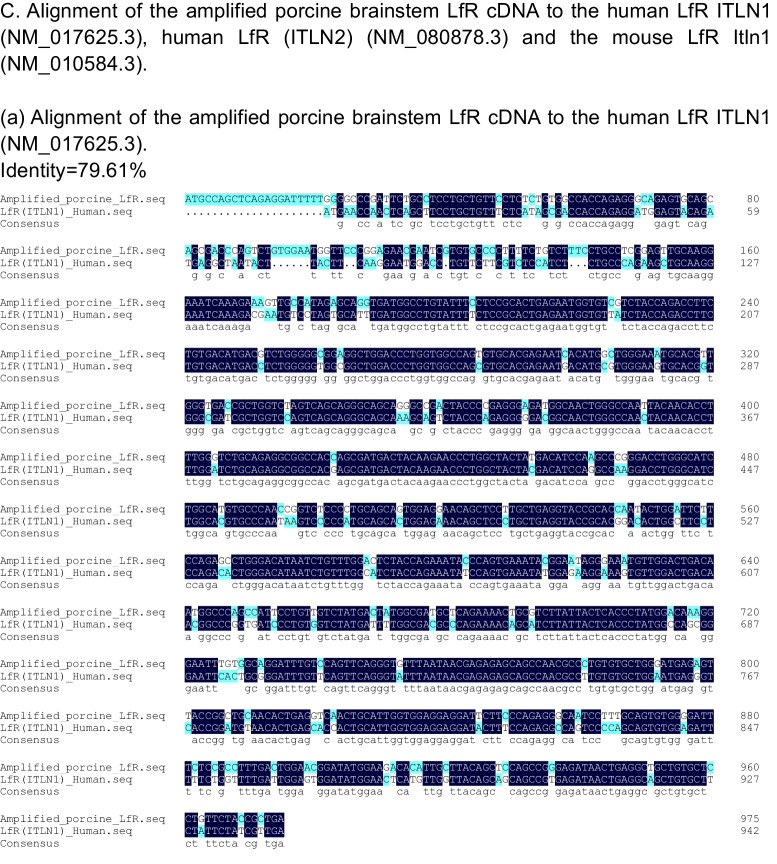

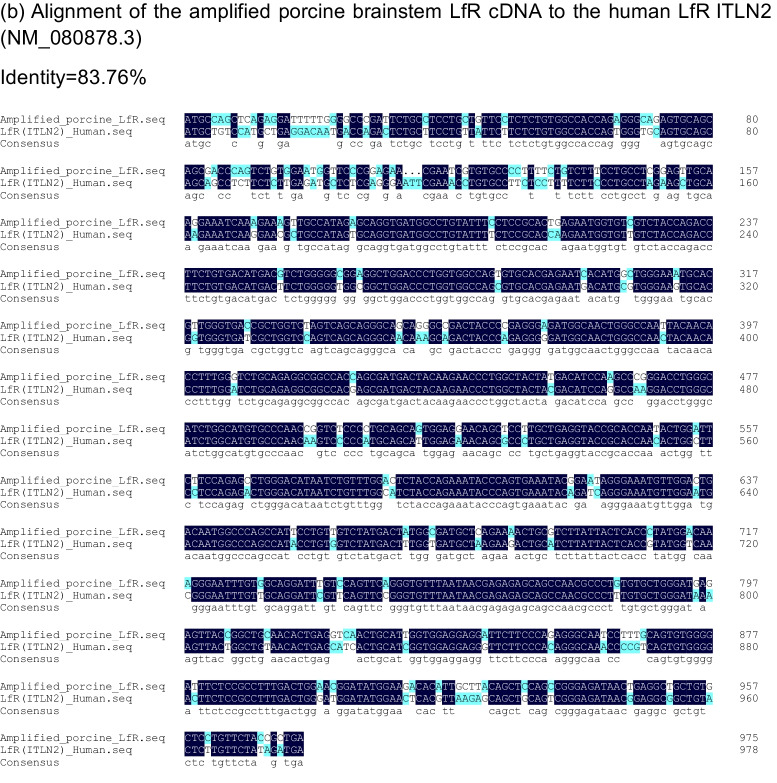

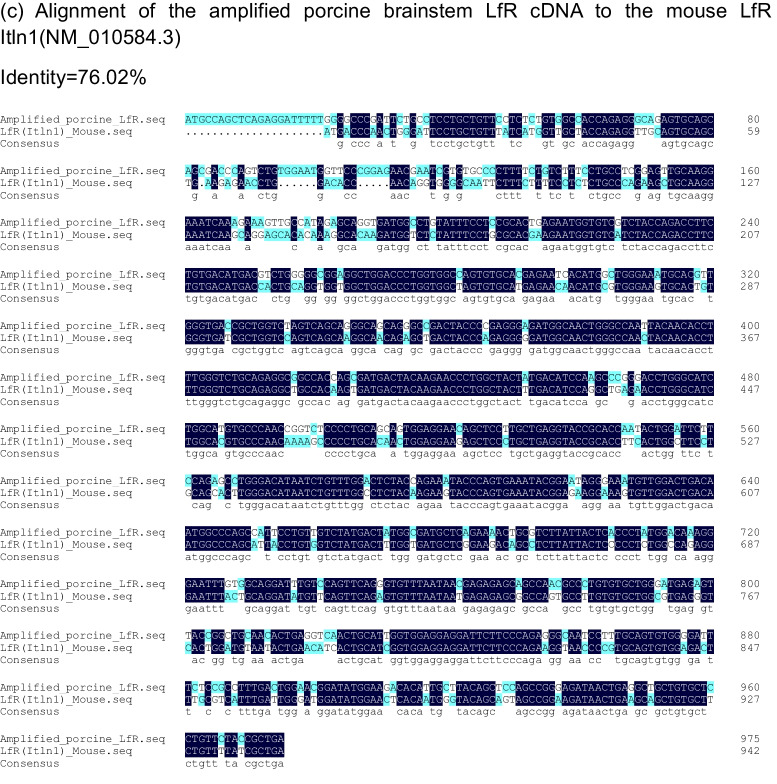
Sequence and structural features of the piglet lactoferrin receptor. **A** Comparison of the ClustalW alignments of the amplified LfR cDNA sequence to the accession no. AY609994.1 sequence. **B** Comparison of the ClustalW alignments of the amino acid sequence from the amplified porcine brainstem LfR cDNA sequence to the amino acid sequence from the accession no. AY609994.1 sequence. **C** Alignment of the amplified porcine brainstem LfR cDNA to the human LfR ITLN1 (NM_017625.3), ITLN2 (NM_080878.3), and mouse LfR Itln1(NM_010584.3) sequences, respectively

The LfR has been reported in porcine intestine [23], but there is no information on LfR expression in porcine brain. Further, the LfR has been cloned and studied in several mammalian species including humans and rodents [17, 23, 35, 36], but only one study reported the cDNA sequence for LfR in piglet small intestine [23]. To understand the molecular mechanisms underlying the role of LfR in brain function, we further cloned and characterized the LfR gene in porcine brain. To this end, a coding sequence with a length of 972 bp that encoded 324 amino acids was obtained and characterized by the cDNA sequence for LfR in the porcine brainstem aligning with the online porcine sequence (GenBank AY609994.1) (Fig. 3A). This alignment showed that there was 98.9% and 98.1% identity in the nucleotide and amino acid sequence with GenBank AY609994.1, respectively (Fig. 3A & B). Furthermore, results showed that the amplified porcine LfR nucleotide sequence had 86.9% identity with the available human LfR ITLN1 (NM_017625.3), ITLN2 (NM_080878.3), and mouse LfR Itln1(NM_010584.3) sequences (Supplementary Fig. 3), of which 79.6% and 83.8% identity was identical to the human sequences and 76.0% was identical to the mouse sequence (Fig. 3C).

### Gene Expression Profile of LfR in Porcine Brain

**Fig. 4:**
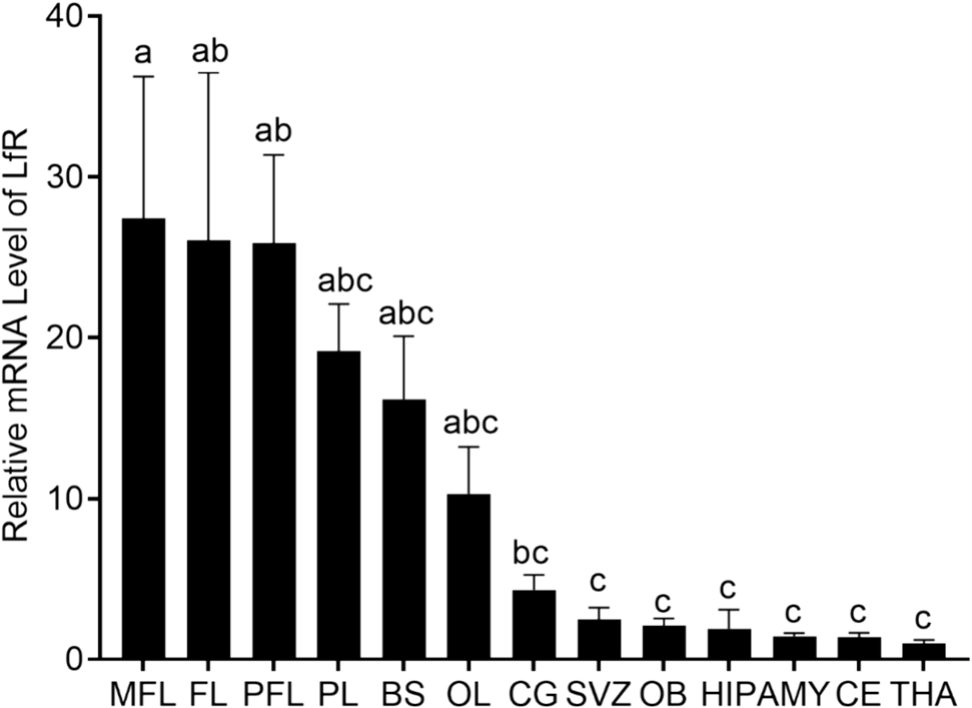
Comparison of LfR gene expression in the different brain anatomical regions of the piglets at 39 days of age. Levels of LfR gene expression in the THA were used for reference (1). Data represent mean ± SEM, *n* = 4 per anatomical region. The overall differences in mRNA levels were significantly different (*p* = 0.00001) based on the two-way ANOVA analysis. Columns bearing different letters within a group were significantly different (*p* < 0.05) based on the general linear model [univariate analysis of variance (ANOVA)] with Bonferroni’s adjustment for multiple comparisons. MFL (middle frontal lobe), CG (cingulate gyrus), SVZ (subventricular zone), OB (olfactory bulb), HIP (hippocampus), AMY(amygdala), THA (thalamus), FL (prefrontal lobe), PFL (posterior frontal lobe), PL (parietal lobe), BS (brainstem), OL (occipital lobe)

While LfR plays an important role in absorption of LF and LF-bound iron, there is no information on the gene expression profile of LfR in the brain. To better understand the functions of LF in neurodevelopment, we carried out studies to evaluate the gene expression profile of LfR in control piglets brain 39 days after birth (Fig. 4). To ensure a high amplification efficiency, gene-specific primers were designed, and amplification efficiency was determined for each gene evaluated including the housekeeping genes. The amplification efficiency of LfR, HPRT1, and GAPDH was 98.8%, 93.0%, and 94.9%, respectively (Supplementary Fig. 2).

LfR gene was expressed in different brain anatomical regions studied, with the highest expression levels in the frontal lobe including prefrontal lobe, middle frontal lobe, and posterior frontal lobe, followed by parietal lobe, brainstem, occipital lobe, the cingulate gyrus, subventricular zone, olfactory bulb, hippocampus, amygdala, cerebellum, and thalamus (Fig. 4). In the thalamus, the expression level of LfR gene was 27-fold lower than in the frontal lobe. The overall differences in mRNA expression of LfR between different brain regions were statistically significant (main effect *p* = 0.00001, Fig. 4). Therefore, different regions of the brain perform different neurological functions and have different LfR mRNA levels.

### LfR Gene Expression in Different Regions of the Piglet Brain Responds to LF Intervention

**Fig. 5:**
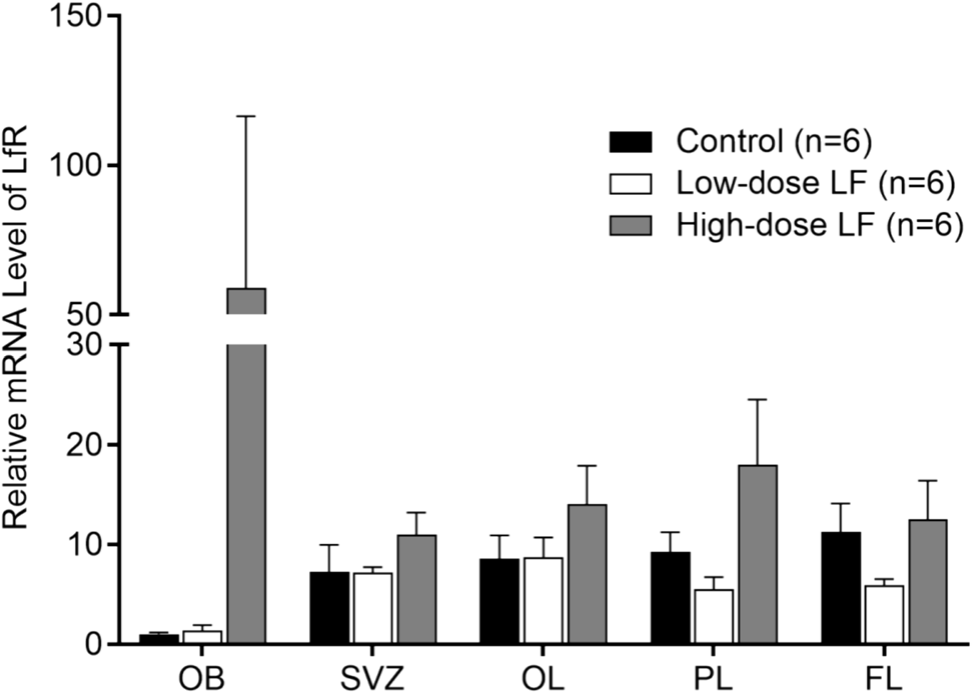
Comparison of LfR gene expression levels (mean ± SEM) in different brain regions between the treatment groups and the control in piglets at 39 days of age. Levels of LfR gene in the OB of the control group were used for reference (1). The general linear model [univariate analysis of variance (ANOVA)] with Bonferroni’s adjustment for multiple comparisons. OB (olfactory bulb), SVZ (subventricular zone), OL (occipital lobe) and PL (parietal lobe), FL (prefrontal lobe)

To identify whether the LfR is involved in the function of dietary LF, 3-day-old piglets were fed different doses of LF for 35 days, and gene expression of LfR in various brain regions was determined at 39 days of age (Fig. 5). In the olfactory bulb, there was a 59-fold increase in the expression of LfR in the high LF group compared to controls suggesting that dietary LF intervention led to increased LfR expression in distinct regions of the brain, although the statistical analysis did not reach significance (*p* > 0.05). There was no significant difference in LfR gene expression in the subventricular zone, occipital lobe, parietal lobe, and prefrontal lobe among the groups (*p* = 0.112–0.387, Fig. 5).

### Protein Expression of LfR in Different Regions of Piglet Brain Responds to Different Doses of LF Intervention

**Fig. 6:**
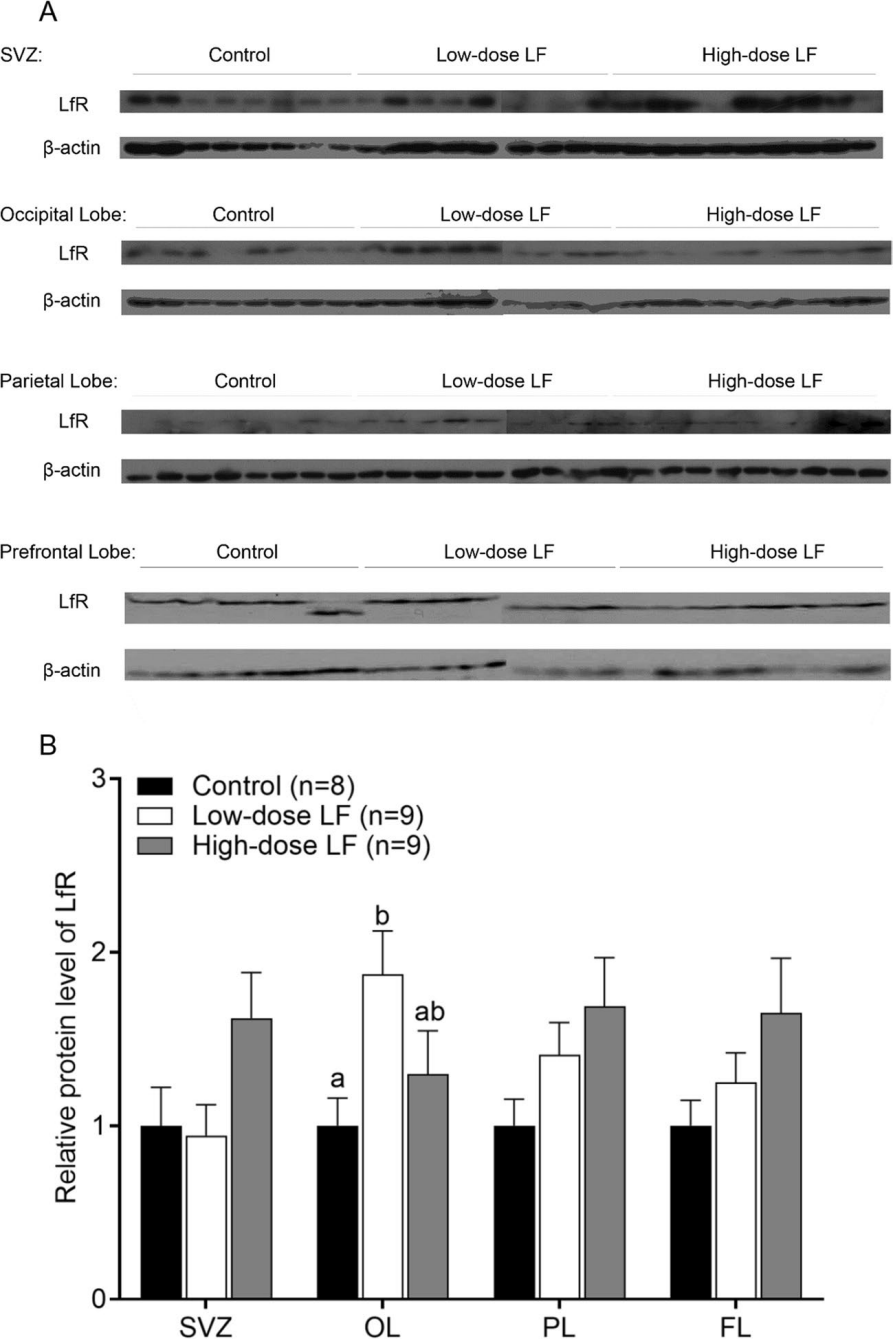
LF supplementation up-regulated LfR protein expression (mean ± SEM) in selected regions of the brain in postnatal piglets. **A** Western blot images of LfR protein in the subventricular zone (SVZ), occipital lobe (OL), parietal lobe (PL), and prefrontal lobe (FL) of piglet between the low dose, high dose of LF, and the control group. **B** Immunohistochemistry analyses of the abundance levels of LfR protein in the different tested brain regions. Levels of LfR protein in the control group were used for reference (1). The overall LfR protein expression between the groups was significantly different (*p* = 0.039) in the occipital lobe based on the two-way ANOVA analysis. Columns bearing different letters within a group were significantly different (*p* < 0.05) based on a general linear model (univariate ANOVA) with Bonferroni’s adjustment for multiple comparisons

Next, we examined whether the expression of LfR protein was affected by dietary LF. Protein expression of the LfR also significantly increased with increasing LF in the diet (Fig. 6). Piglets receiving the high LF diet had a 39% and 41% higher LfR protein expression compared to the control group in the prefrontal lobe (FL) and parietal lobe (PL), respectively, but this comparison between the high vs the low LF group was 17% and 24%, respectively. Thus, a trend dose response in the expression of LfR was observed in prefrontal and parietal lobe, but the overall difference among the groups did not reach significance (main effect *p* = 0.110–0.154). The expression of LfR protein in the subventricular zone (SVZ) of the high LF group was 42% and 38% higher than that of the low LF group and the control group, respectively, but the overall statistical analysis between the groups was marginally significant (main effect *p* = 0.076). The results infer that only the high LF intervention promoted LfR expression in the SVZ. Interestingly, a unique trend of the highest expression of LfR protein in the occipital lobe was the low LF group and then the high LF group and the control group, with an overall significant difference among the groups (main effect *p* = 0.039, Fig. 6). In addition, the expression level of LfR protein in the occipital lobe of the low LF group was significantly higher than that of the control group (*p* < 0.05), but there was no difference for the high LF group (*p* > 0.05) in the Bonferroni post hoc tests analysis. Overall, the results at the protein level confirm our findings at the mRNA level and suggest that the expression of LfR is influenced by dietary LF.

### Working Memory of Piglets Responds to Different Doses of LF

**Fig. 7:**
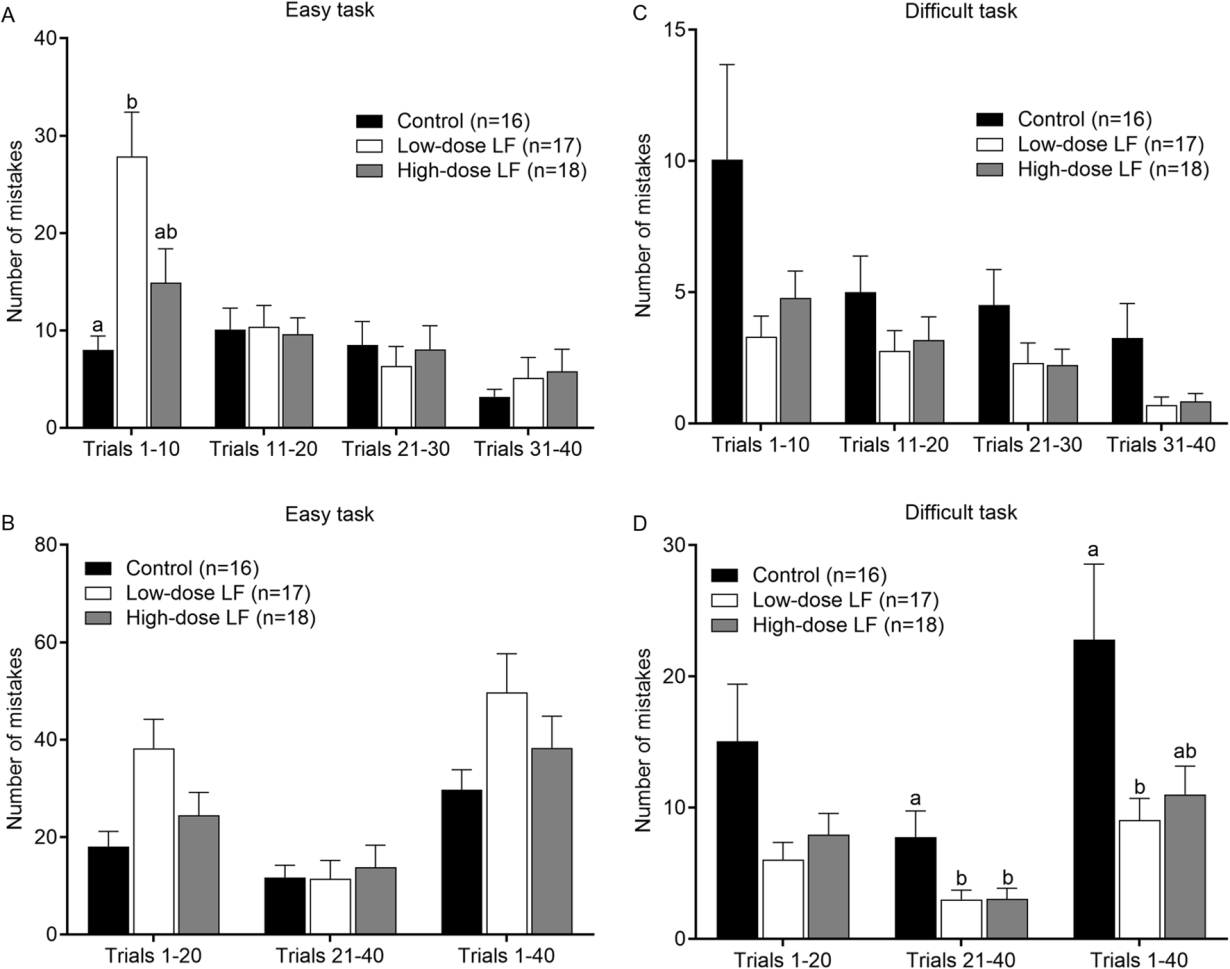
Assessment of the piglets’ working memory in the control and low- and high-dose LF supplemented group in piglets. Number of mistakes made during different stages (per 10 trials) in the easy (**A**) and difficult (**C**) tasks for working memory performance. Each 20 trials and the total 40 trials of the easy tasks (**B**) and the difficult tasks (**D**) were evaluated. Data represent mean ± SEM. The general linear model [univariate analysis of variance (ANOVA)] with Bonferroni’s adjustment for multiple comparisons. *p < 0.05. *A Overall differences between the groups (*p* = 0.032), low dose vs the control (*p* = 0.036), Bonferroni’s post hoc test. *C Overall differences between the groups (*p* = 0.040). *D In the trials 21–40, overall differences between the groups (*p* = 0.016), the control vs the low and high dose *p* = 0.036, Bonferroni’s post hoc test, and in the trials 1–40, overall differences between the groups (*p* = 0.019), the control vs the low dose *p* = 0.027, Bonferroni’s post hoc test

We examined whether the working memory of piglets was affected by dietary LF. We considered the total number of mistakes that the piglets made repeatedly by entering the same wrong doors as a measure of working memory. In the easy task, the total number of mistakes in the low LF group was significantly higher than in the control group (*p* < 0.05, Fig. 7A), but not different from the high LF group in the first 10 trials, although the overall difference among the three groups was statistically significant (main effect *p* = 0.032, Fig. 7A). There was no significant difference in the total number of mistakes among the three groups from the 2nd to the 4th 10 trials of the easy task (Fig. 7A). We also considered that the first 20 trials of the easy task were likely to be mostly “trial and error” and therefore analyzed trials 21–40 or 1–40 separately, but there were no significant differences between groups (Fig. 7B).

For the difficult task, the total number of mistakes in the low and high LF groups was threefold and twofold lower than in the control group for the first 10 trials, respectively, and the overall difference among the three groups was marginally significant (main effect *p* = 0.074, Fig. 7C). For the second and third 10 trials, the total number of mistakes in the two LF supplemented groups was 37–51% lower than in the control group (Fig. 7C), but the difference among the groups was not significant (*p* > 0.05). In the fourth set of 10 trials, however, the control group made 4.6-fold and 3.9-fold more mistakes than the low and high LF groups, respectively (*p* < 0.05, Fig. 7C). In addition, when we analyzed the combined 1–20 and 21–40 trials separately, the control group made 2.5-fold and 1.9-fold more mistakes than the low and high LF groups in the 1–20 trials (main effect *p* = 0.057) and 2.6-fold and 2.5-fold more mistakes in trials 21–40, respectively (Fig. 7D); the difference among the groups was significant (main effect *p* = 0.016). The total number of mistakes in the control group was significantly higher in the working memory test compared to the low (2.5-fold) and high LF groups (2.1-fold), respectively, when we analyzed the combined 1–40 trials (main effect *p* = 0.019, Fig. 7D). Therefore, the two LF supplemented groups exhibited significantly better working memory compared to the control group, but no dose–response relationship for the difficult task.

Because the piglets would have used learning in the easy task to aid learning in the difficult test, we considered the total number of mistakes of working memory made in the easy task as a covariate of working memory for the difficult task. The difference among the groups remained highly significant at all stages of the working memory tests (main effect *p* = 0.019–0.04), and no dose response was observed. Thus, we confirmed that LF supplementation significantly improved working memory ability for the difficult task, but there was no dose response.

## Discussion

LF is a multifunctional glycoprotein and is widely distributed in various tissues and external secretions [37]. Oral administration of LF has been shown to improve neurocognitive ability by upregulating the BDNF signalling pathway and polysialylation with a lower concentration of Lf enhancing neurodevelopment and cognition and a higher concentration being more neuroprotective [6, 8]. To demonstrate whether LfR in the brain responds to LF supplementation and contributes to brain development and cognition, piglets were chosen as an animal model, because its brain structure and function closely resemble that of human infants [21, 38]. Further, pigs, like humans, have LfRs throughout their small intestine [23]. We first determined the cDNA sequence for LfR in the brainstem of piglets and aligned it with the reported human ITLN1 (NM_017625.3) and ITLN2 (NM_080878.3) and mouse sequences (Itln1, NM_010584.3) to provide a better understanding of the identity and conserved regions of LfR across species (Fig. 3A, B, C). Our results confirmed the amplified sequence as that of LfR and validated the use of specifically designed primers to target LfR expression in porcine brain.

Subsequently, we determined the relative expression profile of LfR in various regions of the piglet brain by quantitative RT-PCR. The expression of LfR mRNA was highest in the frontal lobe including the prefrontal lobe, middle frontal lobe, and posterior frontal lobe compared to the other 10 examined brain regions (Fig. 4). The frontal lobe is the largest of the four major lobes of the brain in mammals and plays a key role in retrieving text and visual explicit memories into the working memory and forming long-term memories [39]. Further, most of the brain regions examined are related to memory function, e.g., the hippocampus, a structure of the medial temporal lobe for memory processing, learning, spatial navigation, and emotions [40]. Our results suggest that different regions of brain express different levels of LfR mRNA, and that LF, acting through binding to LfR in different brain regions, may be associated with its specific functions of neurodevelopment [6, 7], neuroprotection [7, 41, 42], and learning and memory [6, 7, 37]. The lowest expression of LfR in the thalamus suggests that only a relatively low level of LfR expression is actually required for its function. The thalamus is the relay station of the brain. A large amount of information (except for olfaction information) that reaches the cerebral cortex for interpretation must be processed through the thalamus [43].

We determined the effect of different doses of dietary LF on the transcription level and posttranslational expression of LfR in different brain regions. At the transcriptional level, the expression of LfR in distinct regions of the brain increased with increasing LF concentration in the diet. It is notable that LfR mRNA in the olfactory bulb did not significantly respond to the low LF intervention, but high LF dramatically increased LfR gene expression by 59-fold, although this was not significant (*p* > 0.05, Fig. 5) compared with the control group. The olfactory bulb plays a central role in the processing of olfactory information, which directly routes to the amygdala and hippocampus and is related to emotion, memory, and learning. The mRNA and protein levels of LfR in the subventricular zone (SVZ) were increased by 34% and 38%, respectively, in the high LF group compared to the control group, but the overall comparison between the groups was not significant with respect to mRNA and marginally significant with respect to its protein (Figs. 5 & 6). Low LF diet did not significantly affect mRNA or protein expression of LfR in the SVZ region. Thus, increased expression levels of LfR in the olfactory bulb and SVZ brain regions may require high LF intake. The SVZ of the dentate gyrus of the hippocampus is one of the major brain regions maintaining neurogenesis throughout life. Adult SVZ neurogenesis takes the form of neuroblast precursors of interneurons that migrate to the olfactory bulb through the rostral migration stream [44]. Lf may cross the blood–brain barrier (BBB) from the peripheral circulation through lipoprotein receptor-related protein (LRP)-mediated endocytosis [45] and accumulates in the brain capillary endothelial cells [46]. BBB endothelial cells lining the inner walls of blood vessels facilitate the internalization and transport of bioactive LF across the BBB [45]. In addition, several LF putative receptors, such as low-density lipoprotein receptor-related protein 1 [47] and intelectin-1 [48], are present in brain endothelial cells and promote transcytosis into the brain parenchyma [45]. Increasing evidence also suggests that Lf crosses the BBB and exerts its neuroprotective effects [46, 49]. Furthermore, Lf acts an effective carrier molecule to facilitate transfer of other therapeutic agents across the BBB [50–52]. Whole-genome sequencing analysis showed that the high LF intervention significantly promoted signalling pathways, functions, and networks related to neuroprotective effects [8]. Therefore, LfR may bind LF during cell differentiation to promote neural development. Dietary intervention with high LF may exert neuroprotective effects through LfR. To our knowledge, these findings have not been reported previously.

Dietary LF supplementation increased LfR protein expression levels in all examined brain regions with a trend towards a dose–response relationship in the prefrontal and parietal lobes (Fig. 6). The anatomical structure of the prefrontal lobe controls executive cognitive functions such as future planning, decision-making, memory, and cognitive flexibility. The parietal lobe is a center for sensory perception and integration where the brain interprets input from other areas of the body. Further, both low and high LF intervention significantly increased LfR protein in the occipital lobe of piglets. Studies have shown that piglets learning visual cues in an eight-arm radial maze have significantly improved learning speed and long-term memory when fed low LF diet compared to high LF diet [6, 8]. On the basis of the evidence that brain LfR protein expression responds to dietary LF, it is likely that LfRs were acquired during cell differentiation. Our results thus suggest that dietary LF passes through the BBB and binds its specific receptor, LfR, to enhance visual learning and memory functions. To our knowledge, this finding has not been previously reported.

Finally, we examined working memory performance of piglets in response to low and high LF supplementation. We found that there was a significant increase in working memory performance in both low and high LF supplementation piglets for the difficult task, but not in the easy task, and there was no a dose–response relationships for the easy or difficult tasks (Fig. 7). Intuitively, one might expect the visual cues in the easy task to have been easier to distinguish and learn than those in the difficult task. However, the piglets made less mistakes with the visual cue of the more difficult task. This paradox might be explained by their older age and advancing brain development, but it is also likely that the animals transferred from learning the easy task to the difficult task. Furthermore, in the easy task, the faster learning piglets underwent continuing reinforcement that could have carried over to the more difficult task. Thus, piglets may have leveraged previous working memory from the easy task to help them learn the more difficult visual cue. Not surprisingly, therefore, the total number of mistakes in the easy task of working memory predicted working memory of the difficult task. The overall results were even more significant for all stages of working memory when the number of mistakes in the easy task was taken into account (main effect *p* = 0.019–0.040). When blood plasma cortisol concentration was used as a covariate during learning, no significant effects on working memory outcomes were observed (*p* > 0.05) [8]. Furthermore, the higher expression level of the LfR gene and protein in the different examined brain regions of piglets, the lower the number of mistakes in the working memory test (two-tailed Pearson correlation analysis) when all piglets were included in analyses (*p* > 0.05, supplementary Fig. 5A, B, C, D, E, F, G). However, when we analyzed the data overall comparison of regression line slope among the groups, the two LF supplementation groups were marginally significant for the parietal lobe (*p* = 0.051, supplementary Fig. 5H, *p* > 0.05). LF may enhance working memory in visual discrimination test at the eight-arm maze through a mechanism of binding to its receptor in olfactory bulb.

In this study, dietary intervention with LF had no significant impact on body weight gain throughout the course of the study (Supplementary Fig. 4A), and no significant differences were found in cerebrum weight, cerebellum weight, or the ratio of brain weight to body weight among the three groups at 39 days of age (Supplementary Fig. 4B & C).

In summary, all examined brain regions expressed LfR mRNA and protein, with expression levels varying in specific anatomical regions of the brain. LfR mRNA and protein in different brain regions responded to dietary LF supplementation, but there was no dose response to low and high doses of LF supplementation, except for the prefrontal lobe and parietal lobe. Low and high LF supplementation significantly improved the working memory capacity of piglets for a difficult task but did not show a dose response. The benefits of LF on brain function may be through the mechanism of binding to its receptor. Future studies should investigate the exact relationship and mechanism of LF-LfR in neurodevelopment, neuroprotection, and cognitive functions by overexpression or knockout of LfR in cells and animal models.

## Supplementary Information

Below is the link to the electronic supplementary material.

Supplementary file1 (DOCX 6778 KB)

## Data Availability

Data presented in this study are available on request from the corresponding author.
