# Supplementary material for: Molecular Characterization and Expression of Lactoferrin Receptor (LfR) in Different Regions of the Brain Responding to Lactoferrin Intervention
Source: Mol Neurobiol. 2024 Aug 24;62(3):2857–71. doi: 10.1007/s12035-024-04378-z (PMC11790811; doi:10.1007/s12035-024-04378-z)
Supplement: Supplementary file 1 — Supplementary file1 (DOCX 6778 KB) [file 12035_2024_4378_MOESM1_ESM.docx]

**Supplementary Figure**

**Figure S1.**

**
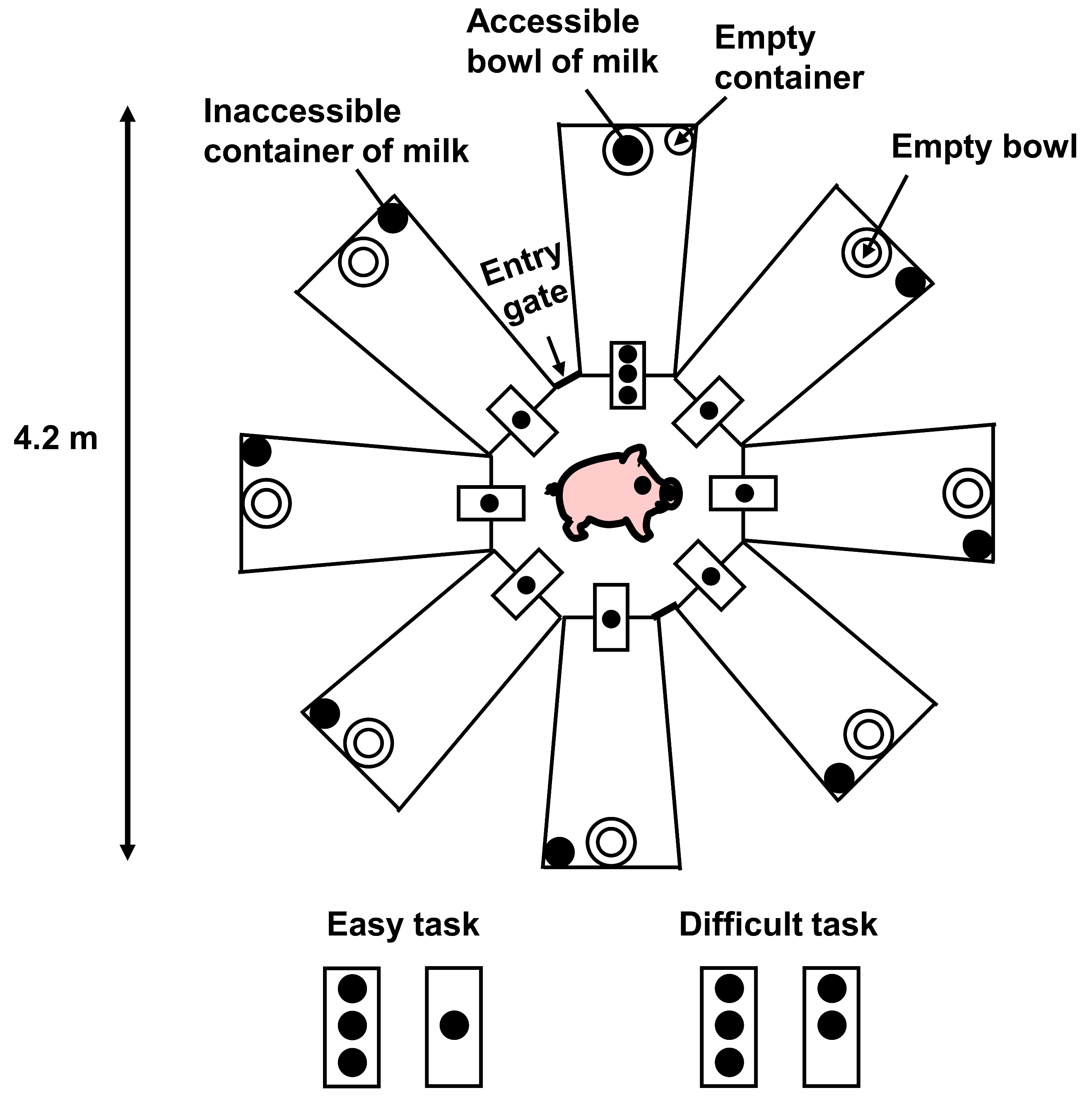
**

**Figure S1.** Schematic diagram of the learning area within the 8-arm radial maze and the visual cues used for the easy and difficult learning tasks. Figure cited from Wang et al. 2007 Am J Clin Nutr.

**Figure S2.**

**(A).**

**
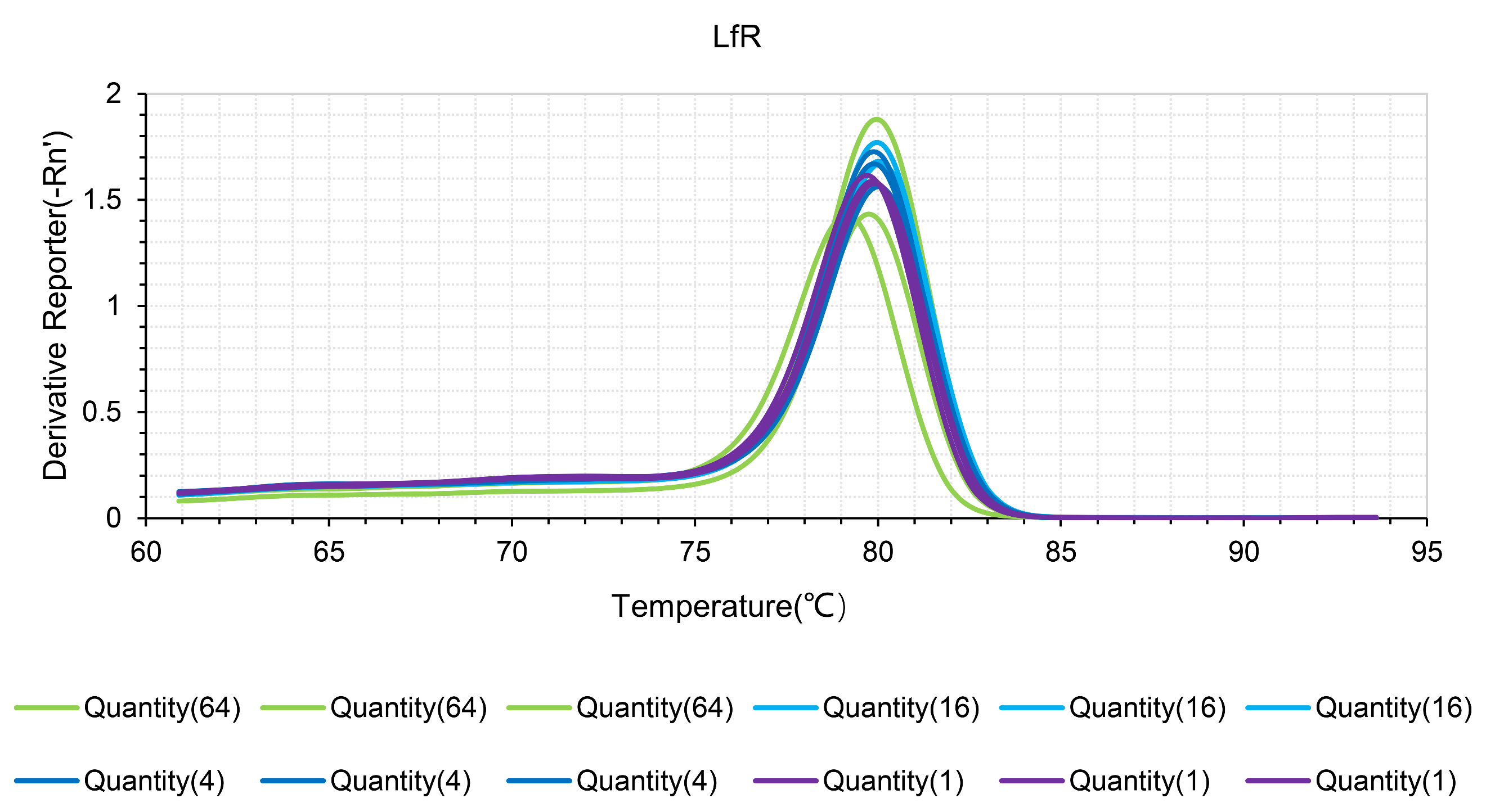
**

**(B).**

**
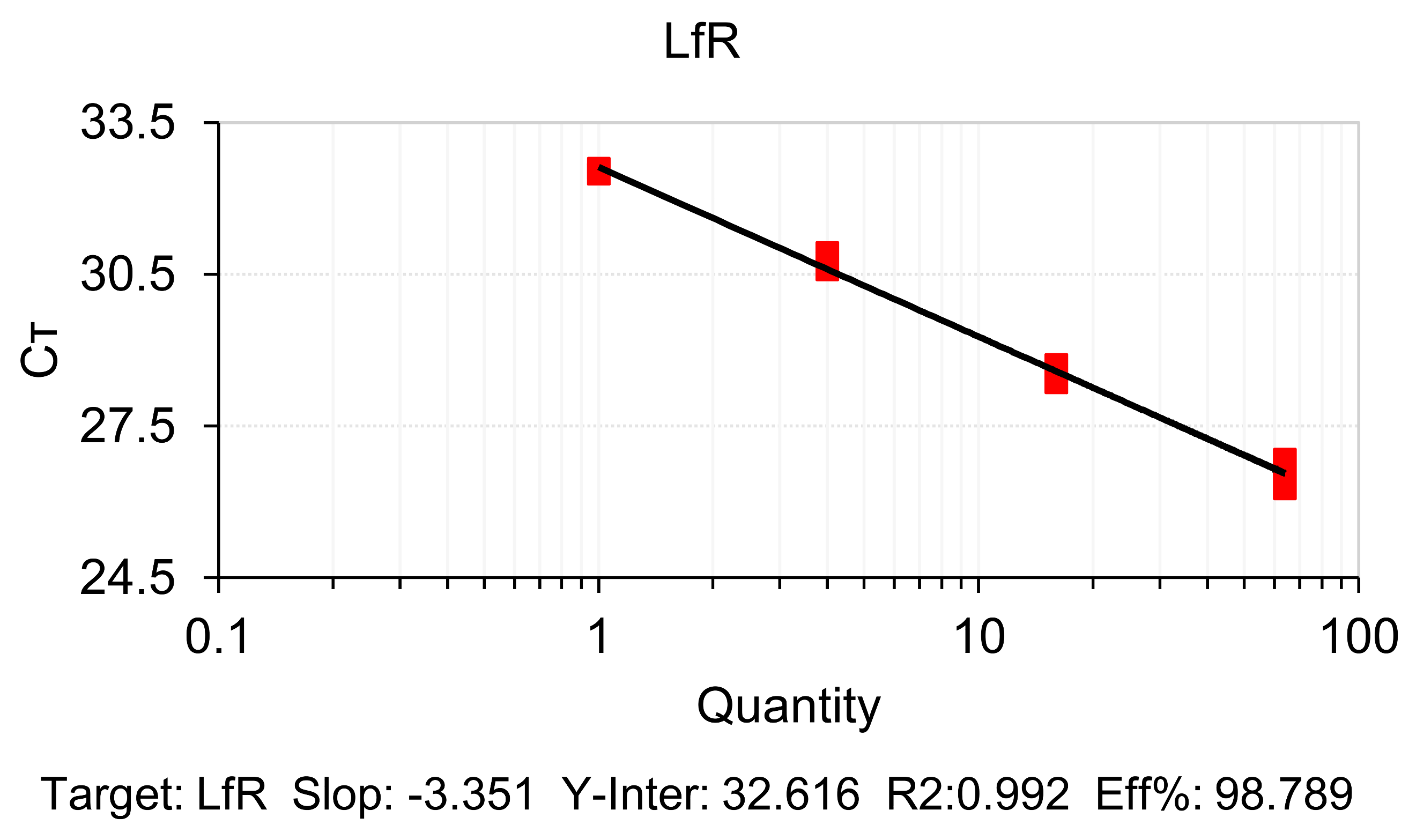
**

**(C).**

**
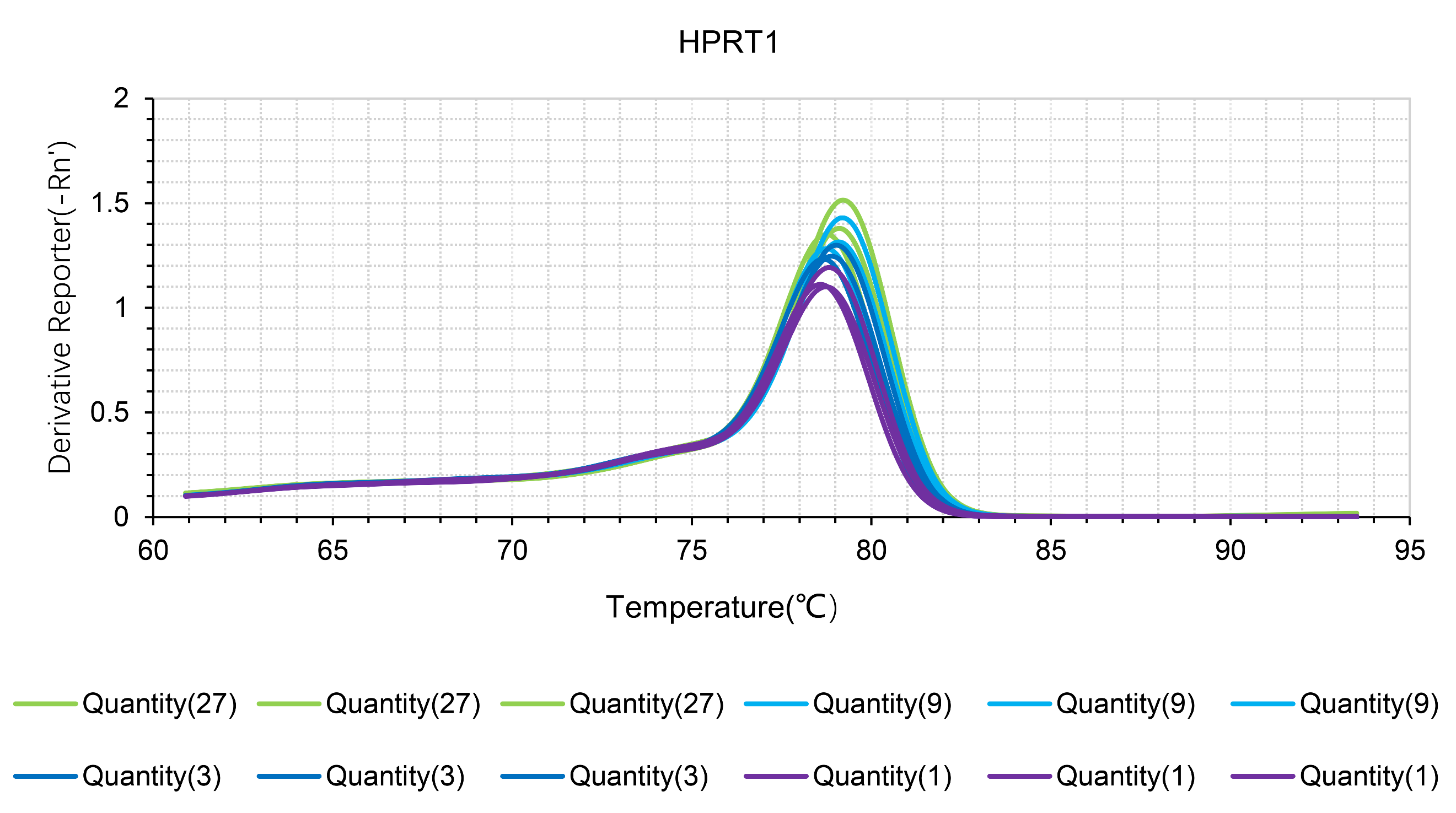
**

**(D).**

**
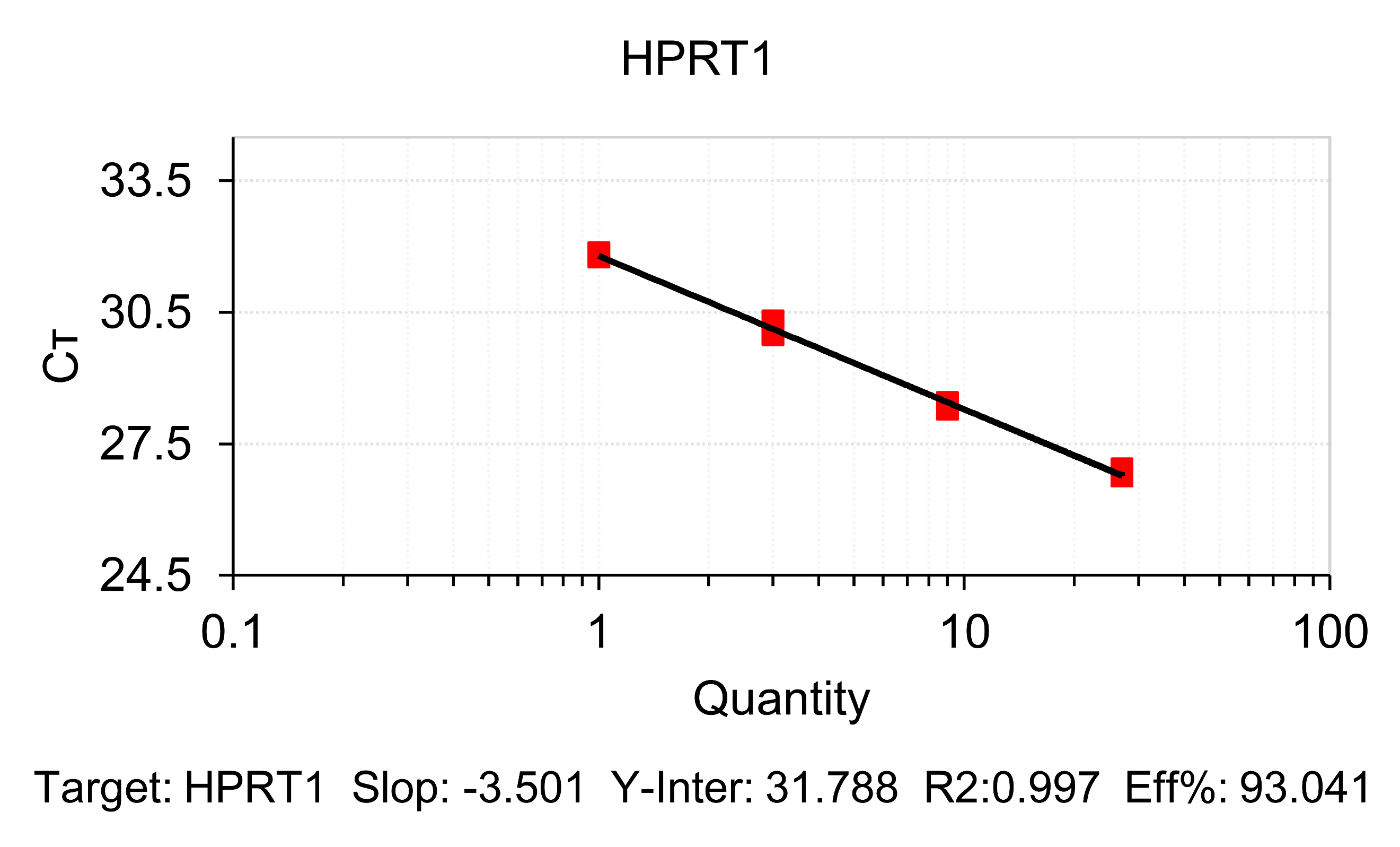
**

**(E).**

**
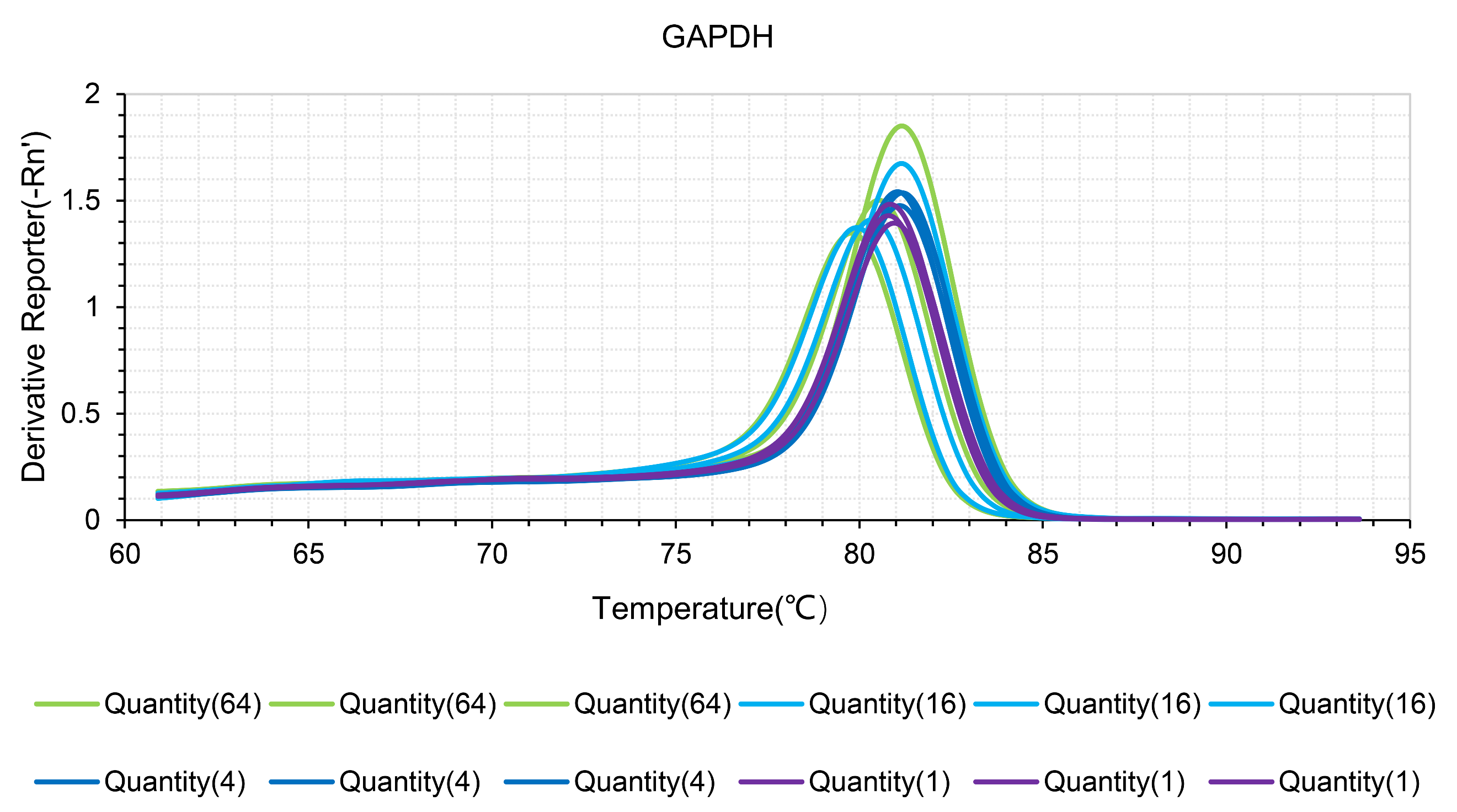
**

**(F).**

**
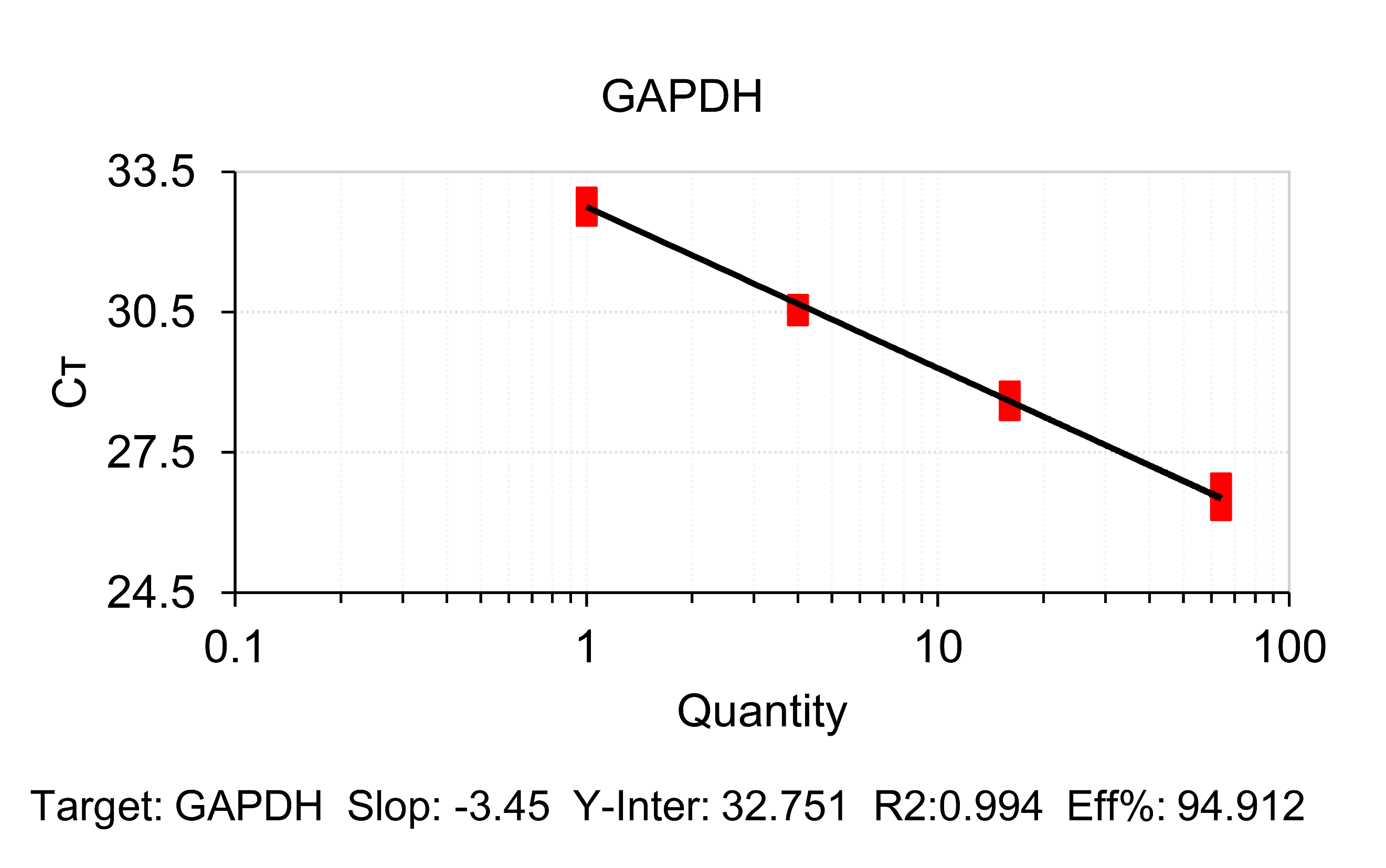
**

**Figure S2.** The primers’ Melt curve and standard curve of LfR, HPRT1 and GAPDH. The primers’ melt curve of LfR (A), HPRT1 (C), GAPDH (E), and the primers’ standard curve of LfR (B), HPRT1 (D), GAPDH (F). The amplification efficiency of LfR, HPRT1 and GAPDH was 98.8%, 93.0% and 94.9%, respectively.

**Figure S3.**

**Figure S3.** Alignment of the amplified porcine brainstem LfR cDNA to the human and mouse LfR sequence. The amplified porcine LfR nucleotide sequence have 86.94% identity with the available human LfR ITLN1 (NM_017625.3), ITLN2 (NM_080878.3) and mouse LfR Itln1(NM_010584.3) sequences.

**Figure S4.**

**(A).**


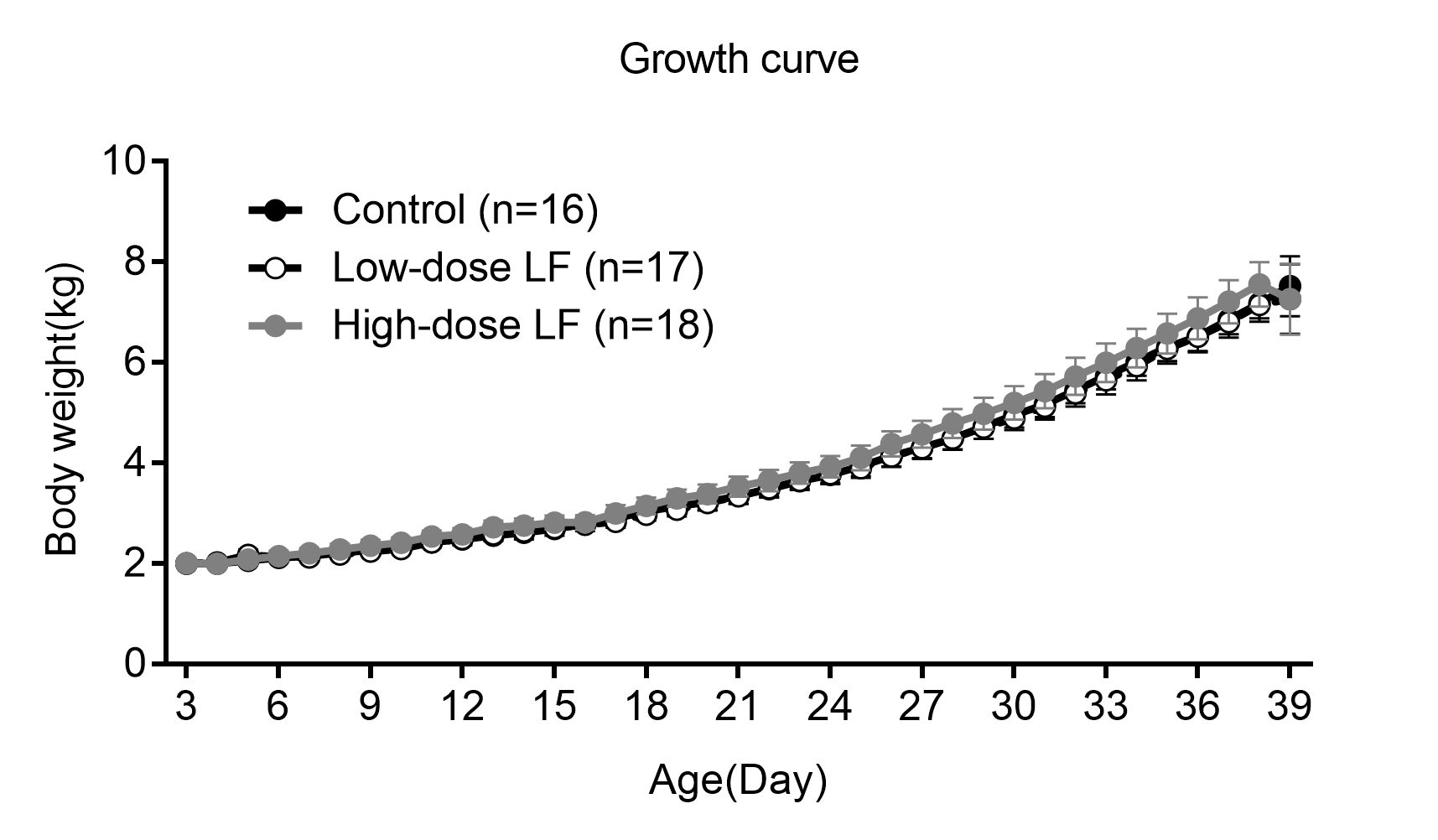


**(B).**


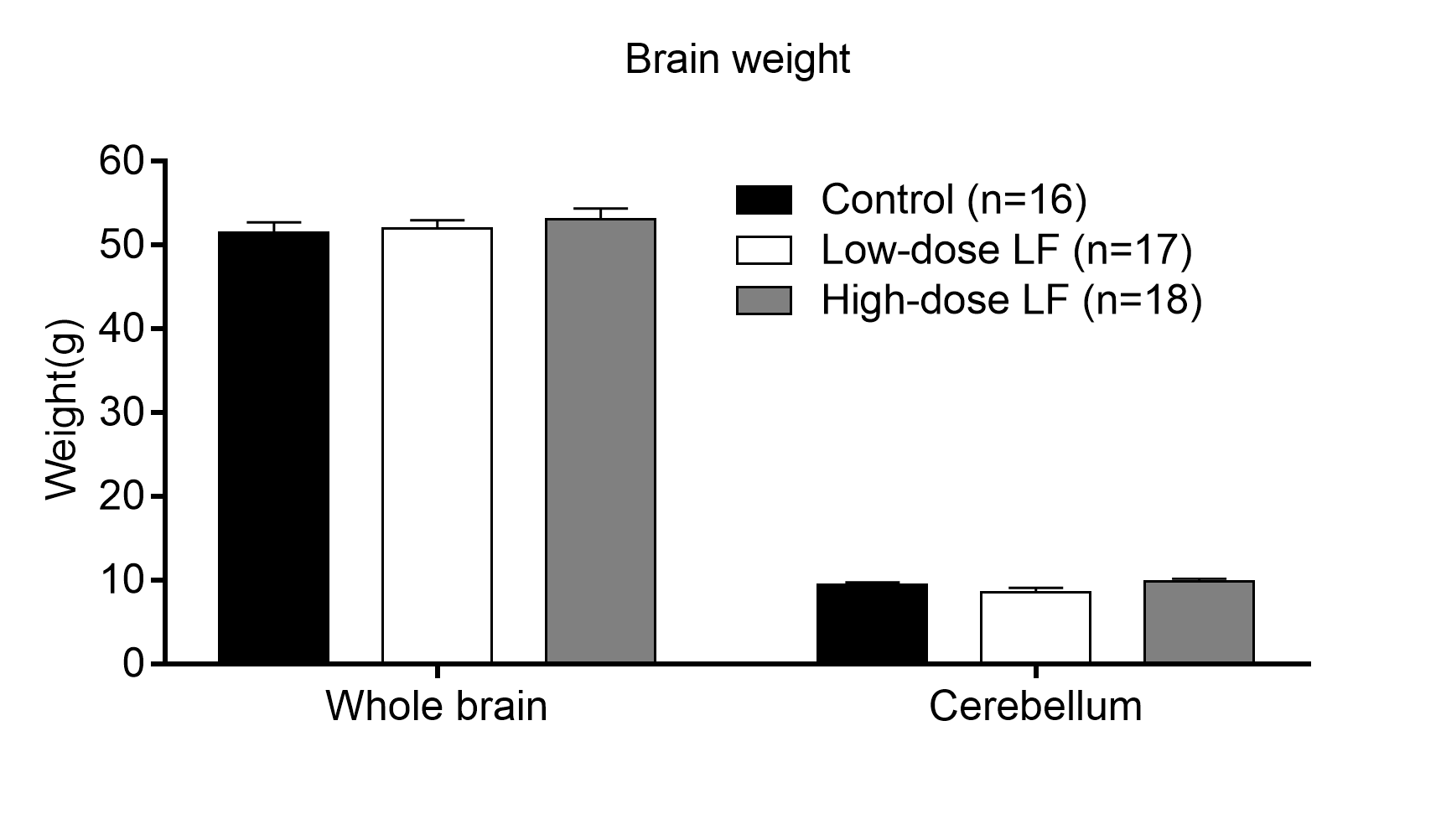


**(C).**


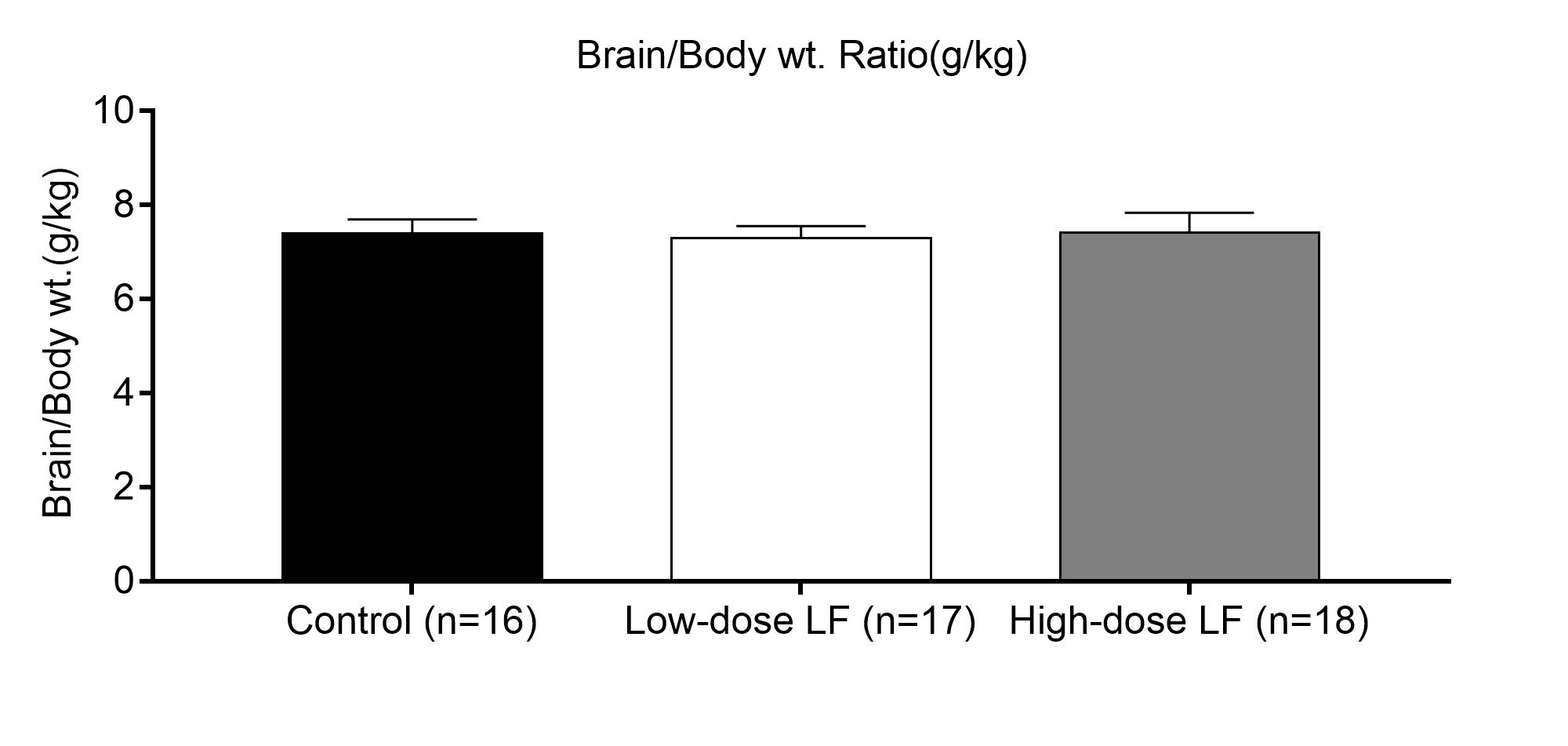


**Figure S4.** The body weight gain of piglets throughout the course of the study (A), the whole brain and cerebellum weight of piglets at 39 days of age (B), and the ratio of brain weight to body weight of piglets at 39 days of age (C).

**Figure S5.**

A





B





C





D





E





F





G





H





I





Figure 5. Total number of mistakes in difficult tasks regressed agaist LfR genes and protein levels expression in different brain regions of piglets. Total number of mistakes in difficult tasks regressed against LfR gene levels: A. Parietal lobe (*p* >0.05), B. Occipital lobe (*p* >0.05), C. SVZ ( *p* >0.05) and D. Olfactory bulb (*p* >0.05), and against LfR protein levels: E. Parietal lobe (*p* >0.05), F. Occipital lobe (*p* >0.05) and G. SVZ (*p* >0.05). H. Overall three groups comparison (regression line slope between the groups) in parietal lobe (low dose vs the control F= 4.612, *p*= 0.051, I. The high dose vs the control F= 2.792, *p*>0.05), Occipital lobe (low -dose vs the control F= 2.500, *p* >0.05; the high dose vs the control F=2.606, *p*>0.05).
